# Practice effect and test–retest reliability of the Wechsler Memory Scale-Fourth Edition in people with dementia

**DOI:** 10.1186/s12877-023-03913-2

**Published:** 2023-04-01

**Authors:** Shu-Chun Lee, Tzu-Hua Chien, Chih-Pang Chu, Yen Lee, En-Chi Chiu

**Affiliations:** 1 Department of Occupational Therapy, Taipei City Hospital, Songde Branch, Taipei, Taiwan; 2 Department of Recreation and Sports Management, University of Taipei, Taipei, Taiwan; 3 Taipei Tzu-Chi Hospital, New Taipei, Taiwan; 4 Division of Psychosomatic Medicine, Taipei City Hospital, Songde Branch, Taipei, Taiwan; 5 School of Education, Edgewood College, Madison, WI USA; 6 Department of Long-Term Care, National Taipei University of Nursing and Health Sciences, No. 365, Mingde Road, Beitou District, Taipei, 112303 Taiwan

**Keywords:** Memory, Practice effect, Reliable change index modified for practice, Reliability, Dementia

## Abstract

**Background:**

The Wechsler Memory Scale-Fourth Edition (WMS-IV) has been widely used to assess memory function in people with dementia. The older adult battery of the WMS-IV includes four indices and seven subtests. The aims of this study were to examine the practice effect and test–retest reliability and calculate the reliable change index modified for practice (RCIp) for the indices and subtests of the older adult battery of the WMS-IV for people with dementia.

**Methods:**

Fifty-six participants completed the WMS-IV twice, two weeks apart. The practice effect was investigated using effect size (Cohen’s *d*) and bootstrapping mixed design analysis of variance while considering the severity of dementia. The test–retest reliability was estimated using intraclass correlation coefficient (ICC).

**Results:**

The results showed non-significant practice effects with Cohen’s *d* < 0.20 in different severities of dementia on two indices and five subtests. The ICC values of these indices and subtests were 0.82–0.85 and 0.57–1.00, respectively. The other two indices (i.e., auditory memory and immediate memory) and two subtests (i.e., logical memory delayed recall and visual reproduction immediate recall) demonstrated small to moderate practice effect (*d* = 0.46–0.74) for people with mild severity of dementia.

**Conclusion:**

On the whole, the WMS-IV has no to moderate practice effects and moderate to excellent test–retest reliability in people with dementia. The values of the RCIp with 95% confidence interval for the indices and subtests were provided in this study, which are useful to clinicians and researchers for interpreting the real score change in persons with dementia. The two indices (i.e., auditory memory and immediate memory) and two subtests (i.e., logical memory delayed recall and visual reproduction immediate recall) with noticeable practice effect should be used with caution when assessing memory function repeatedly in people with mild severity of dementia.

## Introduction

Due to disease and aging, the population experiencing dementia is growing rapidly. The number of people with dementia rose from 57.4 million people in 2019 to 15.8 million people in 2050 [1]. Memory function decline is one of main features for people with dementia and assessment of memory function decline is crucial for clinical diagnosis. Memory function decline influences people with dementia when performing daily tasks [2] and consequently leads to enormous burdens on caregivers [3]. Therefore, a measure of memory function is necessary to aid diagnosis, make treatment plans, and monitor recovery or deterioration of memory function for people with dementia in both clinical and research settings.

The Wechsler Memory Scale-Fourth Edition (WMS-IV) is used worldwide to assess memory function in people with dementia [4]. It contains index scores and subtest scores to describe different aspects of memory function. The WMS-IV has the following three characteristics. First, it assesses visual and auditory memory functions in a comprehensive manner. Second, it includes immediate and delayed memory subscales to verify deficits of short-term memory and long-term memory. Third, it is appropriate for illiterate people, which does not require reading, using only pictures and the recalling of sounded words. Therefore, the WMS-IV is suitable for assessing memory function in people with dementia.

Empirical ground evidence on practice effect and test–retest reliability is essential for clinicians and researchers to ascertain measurement errors in a measure. Practice effect is defined as improvements in test score over repeated administrations, due to earlier experiences in conducting the same items [5]. Reliable change index estimates the change in score regardless of measurement error, which demonstrates a real score change for a person. Reliable change index modified for practice (RCIp) refers to corrected reliable change index while taking practice effect into consideration [5]. Test–retest reliability evaluates the stability of a measure (i.e., same test results over repeated administrations) [6]. Examining practice effect, and test–retest reliability and calculating RCIp are necessary to increase the utility of the WMS-IV in clinical and research settings.

Practice effect and test–retest reliability have been verified as indices of the WMS-IV for healthy people [7]. However, its practice effect and test–retest reliability have not been examined and RCIp has not been calculated in people with dementia, which limits the explanations of the index scores and subtest scores. Therefore, this study aimed to (1) examine the practice effect and test–retest reliability of the indices and subtests of the WMS-IV for people with dementia; and (2) calculate the RCIp values for the indices and subtests. The WMS-IV includes an adult battery (for ages 16–69 years) and an older adult battery (for ages 65–90 years) [8]. We considered the ages of the dementia population and we chose to examine the practice effect and test–retest reliability of the older adult battery for people with dementia in this study.

## Methods

### Participants

We recruited people with dementia who were outpatients from one hospital in northern Taiwan between July 2019 and April 2020. The following criteria were used to determine eligibility in this study: (1) diagnosed as dementia based on the Diagnostic and Statistical Manual of Mental Disorders, fifth edition; (2) aged 65–90 years old (suggested in the WMS-IV manual); (3) stable status (i.e., same scores of the Clinical Dementia Rating [CDR] between two administrations); and (4) willingness to participate in the study (signed informed consent by the patient and family caregiver). The criteria for exclusion were diagnosis of intellectual disability and history of brain injury. This study was endorsed by the Institutional Review Board of the hospital.

### Procedures

One examiner administered the WMS-IV in this study. This examiner was a certified nurse, who had work experience with people with dementia and worked in the cognitive laboratory. The examiner learned and practiced administering and scoring the WMS-IV under a certified psychologist three days a week for two months. People who met the inclusion criteria were assessed by the CDR and WMV-IV twice, two weeks apart. All assessments were conducted in a quiet place to avoid interference and to prevent any effect on participant’s performance. The CDR was administered though interviewing participants with dementia and their caregivers. The demographic data were collected from medical records.

### Measures

The older adult battery of the WMS-IV contains four indices (i.e., auditory memory [AMI], visual memory [VMI], immediate memory [IMI], and delayed memory [DMI]) and seven subtests (i.e., logical memory immediate recall [LM I], logical memory delayed recall [LM II], verbal paired associates immediate recall [VPA I], verbal paired associates delayed recall [VPA II], visual reproduction immediate recall [VR I], visual reproduction delayed recall [VR II], and symbol span [SSP]). The auditory memory and visual memory indices assess the ability of remembering oral and visual information, respectively. The immediate memory and delayed memory indices assess the ability of remembering information presented immediately and 20–30 min delayed, respectively. The LM I subtest assesses immediate narrative memory by immediate free recalling of two short stories. The LM II subtest assesses long-term narrative memory by retelling stories and recognizing questions related to stories. The VPA I subtest assesses immediate verbal memory by immediate cued recalling of 10 word-pairs for four trials. The VPA II subtest assesses long-term verbal memory by recalling and recognizing word pairs. The VR I subtest assesses immediate visual-spatial memory by immediately recalling and drawing five designs. The VR II subtest assesses visual-spatial memory by redrawing and recognizing designs. The SSP subtest assesses visual working memory by recognizing figures and their relative spatial position [8, 9]. The AMI index is summed up by four subtests: LM I, LM II, VPA I, and VPA II. The VMI index is summed up by two subtests: VR 1 and VR II. The IMI index is summed up by three immediate recall subtests: LM I, VPA I, and VR I. The DMI index is summed up by three delayed subtests: LM II, VPA II, and VR II. The age-corrected scaled score of each subtest is on a metric with mean of 10 and standard deviation of 3. The scaled score ranges from 0–19. Each index score is scale on a metric with mean of 100 and standard deviation of 15. The index score ranges from 40–160 [10]. A higher index score and subtest scaled score indicate better memory function.

The CDR is a tool for assessing cognitive and functional impairments in people with dementia. It contains 6 domains: orientation, memory, judgment and problem solving, community affairs, home and hobbies, and personal care [11]. The personal care domain is ranked on a four point scale (0–1-2–3). The other five domains have five grades (0–0.5–1-2–3). The total score is derived from the six domains and defines the severity of dementia: 0 (healthy), 0.5 (earliest cognitive changes, questionable), 1 (mild), 2 (moderate), and 3 (severe). The CDR has adequate reliability and validity in people with dementia [12].

### Data analysis

Effect size (Cohen’s *d*) was calculated to estimate the magnitudes of the practice effects. The criteira of *d* value were: 0.20–0.49, small effect size; 0.50–0.79, moderate effect size; and ≥ 0.80, large effect size [13]. To determine whether the practice effect would differ for participants with two different levels of the CDR (i.e., mild severity of dementia, CDR = 1 and moderate to severe severity of dementia, CDR = 2–3), we applied the bootstrapping mixed design analysis of variance (ANOVA) which can provide reliable conclusions when the data distribution assumption is violated [14]. In the case of an index or subtest whose practice effect was affected by the level of the CDR, we examined practice effect at each level of the CDR using paired *t*-test with bootstrapping. The RCIp with 95% confidence interval (CI) was calculated as follows [5]:

1 $$\mathrm{RCIp with }95\mathrm{\% CI }=\mathrm{ mean practice effect }\pm 1.96 \times \mathrm{ SEdiff},$$

2 $${\text{SE}}{\text{diff}} =\sqrt{\text{2(SEM)}{2}}$$

3 $${\text{SEM}} ={\mathrm{SD}}_{1}\times \sqrt{\text{1-ICC}}$$

where mean practice effect is the mean of the difference between two administrations, SE_diff_ is the standard error of the differences, SEM is the standard error of measurement, SD is the standard deviation of the first administration and ICC is the intraclass correlation coefficient.

The ICC was applied to examine test-rest reliability by a random-effects, two-way analysis of variance. An ICC value of 0.80–1.00 indicates excellent reliability, 0.60–0.79 indicates good reliability, 0.40–0.59 indicates moderate reliability, and < 0.39 indicates poor reliability [15]. ICC values ≥ 0.80 and ≥ 0.90 can be used for group comparions in research settings and for individual comparisons in clinical settings, repectively [16].

## Results

Fifty-six people with dementia completed all assessments. The average age of the participants was 79.4 years and 66.1% were female. The mean score of the CDR was 1.6. The demographic information of the participants is shown in Table 1.

**Table1:** Demographic data of participants (*n*=56)

Characteristic	
Age (years), mean (SD)	79.4 (6.9)
Gender, n (%)	
Male	19 (33.9)
Female	37 (66.1)
Marital status, n (%)	
Unmarried	2 (3.6)
Married	28 (50.0)
Divorced	2 (3.6)
Widowed	24 (42.9)
Education, n (%)	
Elementary school and below	25 (44.6)
Junior high school	8 (14.3)
Senior high school	12 (21.4)
College and above	11 (19.7)
CDR, n (%)	1.6 (0.7)
1	29 (51.8)
2	18 (32.1)
3	9 (16.1)

*SD* standard deviation, *CDR* Clinical Dementia Rating

Different levels of education did not influence the practice effect of the indices and subtests of the WMS-IV. The results of the bootstrapping mixed design ANOVA showed non-significant practice effects on two indices (i.e., VMI and DMI) and five subtests (i.e., LM I, VPA I, VPA II, VR II, and SSP) in different levels of the CDR (*p* = 0.088–0.893) (Table 2). The Cohen’s *ds* of these indices and subtests were < 0.20, except for the VMI index and SSP subtest. The ICC values of the VMI and DMI indices were > 0.80. The ICC value of the VR II subtest was 1.00 and those of the other four subtests were 0.72–0.78.

**Table 2:** Results of practice effect and test–retest reliability (*n* = 56)

Index/subtest	Mean_1_ (SD_1_)	Mean_2_ (SD_2_)	Cohen’s *d*	Mean practice effect (*p* value)	ICC (95% CI)	SEM	SE_diff_	RCIp with 95% CI
Index								
VMI	55.5 (12.5)	58.5 (14.2)	0.23	3.0 (0.520)	0.82 (0.68, 0.89)	5.3	7.5	-11.7, 17.8
DMI	49.7 (8.4)	50.8 (8.4)	0.12	1.0 (0.088)	0.85 (0.75, 0.91)	3.3	4.7	-8.1, 10.2
Subtest								
LM I	2.5 (1.8)	2.8 (2.4)	0.15	0.3 (0.175)	0.73 (0.58, 0.83)	0.9	1.3	-2.3, 3.0
VPA I	1.7 (1.8)	1.8 (1.8)	0.03	0.1 (0.281)	0.72 (0.55, 0.82)	0.9	1.3	-2.6, 2.7
VPA II	1.9 (1.5)	1.9 (1.4)	0.01	0.0 (0.893)	0.78 (0.65, 0.87)	0.7	1.0	-1.9, 1.9
VR II	2.6 (1.2)	2.6 (1.2)	0.00	0.0 (-)	1.00 (1.00, 1.00)	0.0	0.0	0.0, 0.0
SSP	3.5 (2.1)	4.1 (2.6)	0.29	0.7 (0.370)	0.57 (0.36, 0.73)	1.4	2.0	-3.2, 4.6

*VMI* visual memory, *DMI* delayed memory, *LM I* logical memory immediate recall, *VPA I* verbal paired associates immediate recall, *VPA II* verbal paired associates delayed recall, *VR II* visual reproduction delayed recall, *SSP* symbol span, *SD* standard deviation, *ICC* intraclass correlation coefficient, *CI* confidence interval, *SEM* standard error of measurement, *SE*_*diff*_, standard error of the differences, *RCIp* reliable change index modified for practice

The practice effects of two indices (i.e., AMI and IMI) and two subtests (i.e., LM II and VR I) were affected by the different levels of the CDR. Further examination displayed that these indices and subtests had significance in paired *t*-test with bootstrapping and showed small to moderate effect sizes (*d* = 0.46–0.74) for participants with mild severity of dementia (Table 3). The ICC values of these indices and subtests were 0.72–0.76. For participants with moderate to severe severity of dementia, there was no significant practice effect with *d* < 0.20 on the indices and subtests. The ICC values of the AMI index and LM II subtest were ≥ 0.90. The values of RCIp with 95% CI for the four indices and seven subtests are shown in Table 2 and Table 3.

**Table 3:** Results of index and subtest without affecting by different levels of the Clinical Dementia Rating

Index/subtest	Mean_1_ (SD_1_)	Mean_2_ (SD_2_)	Cohen’s *d*	Mean practice effect (*p-*value)	ICC (95% CI)	SEM	SE_diff_	RCIp with 95% CI
Mild severity (*n* = 29)								
AMI	52.1 (11.5)	55.6 (12.0)	0.45	3.4 (0.024)*	0.76 (0.53, 0.88)	5.6	8.0	-12.2, 19.1
IMI	57.4 (12.8)	63.1 (13.6)	0.74	5.8 (< 0.001)*	0.76 (0.36, 0.90)	6.3	8.9	-11.8, 23.3
LM II	2.2 (2.1)	3.0 (2.9)	0.46	0.8 (0.019)*	0.73 (0.48, 0.87)	1.1	1.5	-2.2, 3.7
VR I	4.3 (3.2)	5.8 (3.7)	0.62	1.4 (0.002)*	0.72 (0.38, 0.87)	1.7	2.4	-3.3, 6.2
Moderate to severe severity (*n* = 27)								
AMI	45.4 (4.9)	45.5 (5.3)	0.05	0.1 (0.805)	0.96 (0.91, 0.98)	1.0	1.5	-2.8, 2.9
IMI	43.2 (6.9)	43.7 (8.0)	0.07	0.3 (0.721)	0.84 (0.53, 0.92)	2.8	3.9	-7,3, 7.9
LM II	2.2 (1.3)	2.2 (1.3)	0.00	0.0 (1.000)	0.90 (0.79, 0.95)	0.4	0.6	-1.1, 1.1
VR I	1.7 (1.9)	1.9 (2.1)	0.14	0.2 (0.485)	0.78 (0.57, 0.89)	0.9	1.3	-2.3, 2.7

*AMI* auditory memory, *IMI* immediate memory, *LM II* logical memory delayed recall, *VR I* visual reproduction immediate recall, *SD* standard deviation, *ICC* intraclass correlation coefficient, *CI* confidence interval, *SEM* standard error of measurement, *SE*_*diff*_ standard error of the differences, *RCIp* reliable change index modified for practice

^*^Significant at *p* < 0.05

## Discussion

To the best of our knowledge, this is the first study to examine practice effect and test–retest reliability of both index scores and subtest scores in the older adult battery of the WMS-IV for people with dementia. Considering the severity of dementia, two indices (i.e., AMI and IMI) and two subscales (i.e., LM II and VR I) displayed small to moderate practice effects in people with mild severity. This study provides empirical evidence for enriching the utility of the WMS-IV in clinical and research settings.

The two indices (i.e., AMI and IMI) showed small to moderate effect sizes (*d* = 0.45–0.74) in people with mild severity of dementia, but the two indices demonstrated negligible effect sizes (*d* = 0.05–0.07) in people with moderate to severe severity of dementia. A previous study demonstrated obvious large effect size (η_p_^2^ = 0.33–0.49) on four indices of the older battery for healthy people over a short-term interval [7]. Practice effect is produced when examinees evolve strategies to answer or remember the items from previous experiences [17]. Healthy people without memory deficits and people with mild severity of dementia could evolve strategies and remember items better than people with moderate to severe severity of dementia. Thus, the AMI and IMI indices of the WMS-IV have obvious practice effect in healthy people and people with mild severity of dementia, but these indices demonstrate no practice effect in people with moderate to severe severity of dementia. Regarding to the subtests of the WMS-IV, practice effects were found in two subtests (i.e., LM II and VR I) for people with mild severity of dementia. A possible reason of noticeable practice effect in the LM II subtest could be that examinees received practice using the items in the administration. In the LM I subtest, examinees listened to story A twice and then listened to story B. After 20–30 min, examinees retold stories A and B and were asked questions related to stories A and B in the LM II subtest. Thus, examinees may memorize the stories, especially story A, and thus gains higher scores on the LM II subtest in the second administration. In the VR I subtest, examinees recall and draw five designs. The VR I subtest revealed practice effect maybe because the items were administered from easy to difficult. Examinees could evolve strategies for memorizing figures when being administered items with less difficulty.

Regarding the clinical implication, the values of the RCIp with 95% CI for the indices and subtests were provided in this study. These values are helpful for interpreting the results (i.e., whether a score change for a person with dementia achieves a real deterioration or improvement) with 95% certainty. For example, the value of the RCIp with 95% CI of the AMI index is [-12.2, 19.1] for people with mild severity of dementia. A person with mild severity of dementia having a score change (i.e., posttest minus pretest) lower than -12.2 or higher than 19.1 indicates a real deterioration or improvement, respectively, after intervention. Therefore, the results of this study can support clinicians and researchers in interpreting the scores of a person with dementia more precisely and reasonably, while considering measurement errors, including practice effect.

Satisfactory test–retest reliability of four indices in healthy people [7]. In this study, our findings displayed better test–retest reliability on the VMI and DMI indices in people dementia and the AMI and IMI indices in people with moderate to severe severity of dementia (ICC > 0.80), which can be used for group comparisons. The AMI index with ICC > 0.90 in people with moderate to severe severity of dementia can be applied for individual comparisons. At the subtest level, the SSP subtest showed relatively lower ICC value, which was similar to the test–retest result of the SSP subtest in the Wechsler Memory Scale-Third Edition [18]. Thus, the SSP subtest may not assess particular memory functions consistently over repeated administrations.

Three limitations were noticed in this study. First, participants were recruited from one hospital, which may limit the generalizability of our results. Second, we did not examine the practice effect and test–retest reliability of the adult battery of the WMS-IV in people with dementia. Except for the four indices and seven subtests, the adult battery has one more index (i.e., visual working memory) and one more subtest (spatial addition). Future studies are warranted to examine the practice effect and test–retest reliability of the adult battery for people with dementia. Third, the participants were people with dementia and thus floor effects (i.e., the percentage of participants with lowest score > 20%) were observed in the sample of this study for the indices and subtests of the WMS-IV, except for the VMI index and SSP subtest. The sample size was slightly small and the age-corrected scaled scores of two administrations in the VR II subscale were the same. The small sample size and high floor effect may influence the results of the practice effect and test–retest reliability. Further cross-validation with the big sample size is needed.

## Conclusion

Overall, the WMS-IV has no to moderate practice effects and sufficient test–retest reliability in people with dementia. The values of the RCIp with 95% CI of the indices and subtests in the WMS-IV are provided herein, which can help clinicians and researchers to explain the results of particular memory functions over repeated assessments. The two indices (i.e., AMI and IMI) and two subtests (i.e., LM II and VR I) with obvious practice effect should be used cautiously while repeatedly assessing memory function in people with mild severity of dementia.

## Data Availability

Data supporting the findings are available upon request. Please contact the corresponding author, En-Chi Chiu (enchichiu@ ntunhs. edu. tw), for data access.
